# Transient Headache and Neurological Deficits with Cerebrospinal Fluid Lymphocytosis Associated with IgM Antibodies to the Epstein-Barr Virus Viral Capsid Antigen

**DOI:** 10.1155/2013/975709

**Published:** 2013-04-17

**Authors:** Kossivi Apetse, Ludovic Breynaert, Chloe Butaud, Albert Beschet, Karine Blanc-Lasserre, Loic Ribouillard, Victor Chan

**Affiliations:** Service de Neurologie, Centre Hospitalier de Valence, 179 Boulevard Marechal Juin, 26953 Valence Cedex 09, France

## Abstract

Some authors have suggested that the syndrome of transient headache and neurological deficits with cerebrospinal fluid lymphocytosis (HaNDL) results from an immunological response directed against a viral agent. Here we report a case of HaNDL in an immunocompetent 19-year-old male that could support this hypothesis.

## 1. Introduction

The syndrome of transient headache and neurological deficits with cerebrospinal fluid lymphocytosis (HaNDL) is a benign and rare disease that was first described in the eighties [1, 2]. Some authors have suggested that the HaNDL results from an immunological response directed against a viral agent. We report here a case of HaNDL that occurred in a 19-year-old immunecompetent male who evinced serological evidence of isolated IgM antibodies to the Epstein-Barr virus (EBV) viral capsid antigen (VCA). The simultaneous occurrence of HaNDL and isolated IgM VCA could support the concept that an inappropriate immune reaction underlies the development of the neurological symptoms.

## 2. Case Report

**Figure 1:**
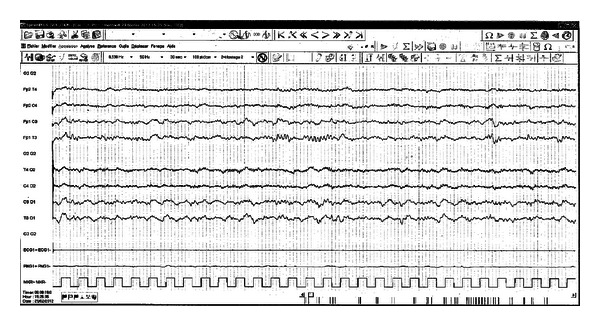
An electroencephalogram showing a nonepileptiform slowing on the left hemisphere in a 19-year-old male HaNDL patient (lines Fp1C3, Fp1T3, C3O1, and T3O1).

A 19-year-old male with no previous history of disease, in particular of migraine, was admitted at Valence Stroke Unit, France, following two episodes of transitory aphasia combined with acute headache and vomiting. This right-hander patient awoke on the day of hospitalization with an unusual frontal, moderate, and nonthrobbing headache combined with expressive aphasia. Clinical examination upon admission one hour later confirmed the moderate headache and one episode of vomiting. In contrast, the aphasic state regressed 20 minutes after its onset. Examination of the throat, spleen, and lymph nodes did not reveal any abnormalities. There was no evidence for an infectious episode, in particular of viral origin, and no sign of recent cranial traumatism. Emergency cerebral MR imaging (including T2*, diffusion-weight, and fluid-attenuated inversion recovery flight images) with intracranial and cervical MR angiography did not reveal any vascular or parenchymal lesions. Upon treatment with intravenous paracetamol, headache diminished in intensity but persisted and the patient kept vomiting. Five hours after onset of the symptoms, the expressive aphasia resumed. Two hours after-relapse of the aphasia, headache and nonfluent speech with reduced vocabulary persisted. There were no other obvious neurological symptoms and the patient was apyretic. An electroencephalogram revealed the presence of nonepileptiform slowing on the left hemisphere (Figure 1) that was consistent with the recorded clinical symptoms. Examination of the cerebrospinal fluid (CSF) found 250 lymphocytes/mL, normal glucose levels, and a minimal increase in protein concentration (0, 54 g/L). A diagnosis of encephalitis caused by herpes simplex 1 (HSV 1) was suspected and the patient was given acyclovir intravenously (10 mg/kg 3 times a day). However, a PCR performed on CSF with primers specific to HSV and EBV delivered negative results. In contrast, EBV serological test (EIA VIDAS, bioMérieux) detected isolated IgM VCA: IgM VCA index = 0, 65, IgG VCA = 0, and IgG EBNA = 0. Serological testing for a recent infection with CMV, VZV, HSV, hepatitis A, B, or C viruses, Lyme borreliosis, rickettsioses, syphilis, or HIV proved to be negative. Peripheral blood counts, C reactive protein, and liver function tests were within normal range. Clinical symptoms disappeared within 24 hours and acyclovir was discontinued upon reception of the negative PCR results, 24 hours later. The patient was discharged after 6 days of hospitalization and was reinvestigated six months later. No neurological signs could be noted with the exception of mild headache. Compilation of the clinical and laboratory features led us to pose a diagnosis of HaNDL. Serological controls at 4 weeks showed an unchanged profile with an IgM VCA index = 0, 31 and negative IgG VCA and IgG EBNA indexes.

## 3. Discussion 

With the notable exception of a multicentric Spanish study that reported 50 cases in 1997 [2], most papers published since the first description of HaNDL were usually restricted to a single patient. These case reports have certainly contributed to the formulation of diagnostic criteria for HaNDL in the International Classification Headache Disorders (ICHD)-II in 2004 [3]. The present case both fulfils these criteria and is in concurrence with the cases reported in the literature.

The precise pathogenesis of HaNDL remains shrouded in obscurity. Multiple hypotheses about potential genetic and/or immunopathological mechanisms induced by an infection have been propounded. The commonalities of clinical symptoms between HaNDL and familial hemiplegic migraine (FHM) have prompted authors to search for common pathogenic mechanisms between these entities. Three gene mutations are constantly found in patients with FHM. The most frequent one involves the CACNA1A gene [4]. However, Chapman et al. could not find this particular mutation in 10 HaNDL patients [5]. Moreover, the absence of HaNDL history or even of migraine in patients with HaNDL does not favour the existence of a predominant genetic predisposition in general. Altogether, if there is any genetic predisposition to HaNDL, it must play a much more limited role than in FHM. 

Various authors have posited that an immunological dysfunction following an infectious episode underlies the pathogenesis of HaNDL for two main reasons: the constant lymphocytosis in the CSF and that the onset of HaNDL was preceded or was concomitant with evidence for an infection. These infectious signs are not always present but are strongly suggestive of HaNDL and have been observed in 20 to 57% of cases [1, 2, 6, 7]. Exhaustive serological screening of neurotropic viruses and PCR-mediated detection are constantly negative. These biological tests are precious to exclude the existence of a meningoencephalitis, the main differential diagnosis of HaNDL. These data suggest that HaNDL results from of an immunological dysfunction induced by an infectious agent, rather than from lesions directly inflicted by a microorganism. Our patient had increased IgM directed against the EBV VCA. These results can be ascribed to an EBV primary infection or to a nonspecific reaction [8]. In the present case, reassessment of the serological profile one month after onset of the symptoms excluded the existence of a primary EBV infection concurrent with the development of the neurological lesions. Therefore, we are left to conclude that we are dealing with a nonspecific false-positive result as has been described during other viral infections [8, 9]. In all probability, our patient recently underwent a viral infection that prompted an episode of HaNDL. We posit that a virus infection elicits an immune response that includes the production of antibodies. The humoral response would be directed against proteins expressed by the vessels that irrigate the dura mater. This would lead to an aseptic inflammation of the trigeminovascular system with, as a consequence [2], (i) the headache, (ii) the transitory defective cerebral perfusion [6] that causes the symptoms of neurological deficits, and (iii) the CSF lymphocytosis. Although likely, such a mechanism would not explain predilection of HaNDL in young male people. Indeed, the diseases that share these pathogenic mechanisms occur at all ages and equally in both sexes; this is, for example, the case of the Guillain-Barre syndrome [10].

## 4. Conclusion 

The HaNDL syndrome remains a differential diagnosis of unclear pathogenesis. Throughout this case report, we have argued about a possible abnormal immune reaction elicited by an infection that would trigger the development of this uncommon affection.
